# Autonomic Nervous System Correlates of Speech Categorization Revealed Through Pupillometry

**DOI:** 10.3389/fnins.2019.01418

**Published:** 2020-01-10

**Authors:** Gwyneth A. Lewis, Gavin M. Bidelman

**Affiliations:** ^1^Institute for Intelligent Systems, The University of Memphis, Memphis, TN, United States; ^2^School of Communication Sciences and Disorders, The University of Memphis, Memphis, TN, United States; ^3^Department of Anatomy and Neurobiology, University of Tennessee Health Sciences Center, Memphis, TN, United States

**Keywords:** pupillometry, categorical perception, speech-in-noise (SIN) perception, listening effort, eye behavior

## Abstract

Human perception requires the many-to-one mapping between continuous sensory elements and discrete categorical representations. This grouping operation underlies the phenomenon of categorical perception (CP)—the experience of perceiving discrete categories rather than gradual variations in signal input. Speech perception requires CP because acoustic cues do not share constant relations with perceptual-phonetic representations. Beyond facilitating perception of unmasked speech, we reasoned CP might also aid the extraction of target speech percepts from interfering sound sources (i.e., noise) by generating additional perceptual constancy and reducing listening effort. Specifically, we investigated how noise interference impacts cognitive load and perceptual identification of unambiguous (i.e., categorical) vs. ambiguous stimuli. Listeners classified a speech vowel continuum (/u/-/a/) at various signal-to-noise ratios (SNRs [unmasked, 0 and −5 dB]). Continuous recordings of pupil dilation measured processing effort, with larger, later dilations reflecting increased listening demand. Critical comparisons were between time-locked changes in eye data in response to unambiguous (i.e., continuum endpoints) tokens vs. ambiguous tokens (i.e., continuum midpoint). Unmasked speech elicited faster responses and sharper psychometric functions, which steadily declined in noise. Noise increased pupil dilation across stimulus conditions, but not straightforwardly. Noise-masked speech modulated peak pupil size (i.e., [0 and −5 dB] > unmasked). In contrast, peak dilation latency varied with both token and SNR. Interestingly, categorical tokens elicited earlier pupil dilation relative to ambiguous tokens. Our pupillary data suggest CP reconstructs auditory percepts under challenging listening conditions through interactions between stimulus salience and listeners’ internalized effort and/or arousal.

## Introduction

Virtually all sensory signals vary along a physical continuum, yet, we tend to perceive them as discrete perceptual objects. Such categorical perception (CP) deciphers meaningful patterns in complex sensory input by organizing information into coherent groups (equivalence classes) (Goldstone and Hendrickson, 2010). Nowhere is this phenomenon more robustly demonstrated than in speech perception. When listeners hear tokens from a phonetic continuum, their discriminability is very good for sounds straddling the category boundary near the midpoint, but very poor for sounds on the same side (Liberman et al., 1967; Pisoni, 1973; Harnad, 1987; Pisoni and Luce, 1987; Bidelman et al., 2013). CP streamlines speech processing by emphasizing acoustic contrasts between- rather than within- phoneme categories (Myers and Swan, 2012), presumably by weighting cues for comparison against internalized templates of a person’s native speech sounds (Kuhl, 1991; Iverson et al., 2003; Guenther et al., 2004; Bidelman and Lee, 2015).

Neuroimaging work has revealed neural processes leading up to categorical decisions (Sharma and Dorman, 1999; Binder et al., 2004; Chang et al., 2010; Zhang et al., 2011; Bidelman et al., 2013; Bidelman and Lee, 2015). In the auditory sciences, research has associated measures of perceptual performance and “listening effort,” which is the deliberate allocation of (available) mental resources to overcome goals when carrying out a listening task (for review see, Zekveld et al., 2018). Under the Framework for Understanding Effortful Listening (FUEL), listening effort is determined by the combined effect of input-demands (e.g., signal quality) and internal factors (e.g., arousal, attention, and motivation) (Pichora-Fuller et al., 2016). Accounting for the latter is crucial interpreting apparent task-related differences.

Diverse experimental techniques have shown that noise degradation has robust consequences for perceptual performance (e.g., Gatehouse and Gordon, 1990), short-term memory performance (e.g., Heinrich et al., 2008), neural activity (e.g., Scott et al., 2000), and pupil reactivity (e.g., Zekveld et al., 2011). Acoustic noise burdens cognitive load, but speech intelligibility is not always straightforwardly predicted by signal-to-noise-ratio (SNR) (for review see, Bidelman, 2017). Under the Ease of Language Understanding (ELU) model (Rönnberg et al., 2013), acoustic input that deviates from a listener’s long-term phonological memory store requires additional cognitive resources for recognition, including working memory and executive functions. The degree to which listeners engage explicit cognitive processes is thought to reflect task-related listening effort, however, cognitive resources and intrinsic motivation may be insufficient for recognition when the mismatch between percept and expectation is too extreme (Ohlenforst et al., 2017).

Segregating a speech signal from acoustic noise is cognitively demanding, drawing on resources for encoding that are normally used for other processes (Cousins et al., 2014). Mechanisms for signal separation might be more readily engaged when category boundaries are particularly noisy (Livingston et al., 1998). Neuroimaging data indicates that the brain processes competing sound streams within the same neural pathways, but devotes more attention to the target stream (Evans et al., 2016). Our recent electrophysiological study found that neural activity was not only stronger for category (unambiguous) relative to non-category (ambiguous) speech sounds but the former was more invariant to noise interference, suggesting CP promotes robust speech perception by “sharpening” category members in noisy feature space (Bidelman et al., 2019b).

Because underlying processes are difficult to measure behaviorally, researchers have assessed listening effort with indirect measurement techniques. For example, eyetracking offers an objective glimpse into real-time speech processing (Ben-David et al., 2011) not captured by behavioral measures and self-reports (Wendt et al., 2016). One non-volitional indicator of cognitive processes is pupil reactivity (pupillometry) (see Naylor et al., 2018). Studies have reported close relations between fluctuations in pupil diameter and underlying neural mechanisms (for review see, Eckstein et al., 2017). Pupil diameter increases with momentary cognitive demands (Kahneman and Beatty, 1966) and correlates closely with neuronal activity from the locus coeruleus, which is the principal brain site for synthesizing norepinephrine (i.e., arousal) (Aston-Jones and Cohen, 2005). Thus, pupil diameter indirectly indicates processes below the threshold of consciousness, which can be modulated by task demands. On a practical note, pupillometry complements other online measures of speech processing, is relatively simple to administer, and can be simultaneously registered with neurophysiological measures (e.g., for review see, Winn et al., 2018).

From the perspective of listening effort, pupillometry is an ideal avenue for investigating the physiological nature and individual differences in speech categorization. Germane to our interests in speech processing, aspects of the pupil response systematically vary with processing load when interpreting languages (Hyönä et al., 1995), speech intelligibility (Zekveld et al., 2010), divided attention during speech listening (Koelewijn et al., 2014), semantic ambiguity (Vogelzang et al., 2016), visual-auditory semantic incongruency (Renner and Wlodarczak, 2017), and pseudoword complexity (López-Ornat et al., 2018). Relevant to this study, researchers have used pupillometry and eyetracking methods to examine how acoustically degraded speech influences listening effort (e.g., Bidelman et al., 2019a; Winn et al., 2015). Findings have been largely consistent: peak pupil dilation and latency systematically increase with decreasing speech intelligibility, but only to the extent that cognitive resources are not overloaded (see section “Discussion”) (Zekveld et al., 2010; Zekveld and Kramer, 2014; Wendt et al., 2016; Ohlenforst et al., 2018). Assessing how pupil responses vary with listening effort could reveal how CP reconstructs auditory percepts under challenging listening conditions. Presumably, speech categorization depends on interactions between stimulus salience (Liao et al., 2016) and listeners’ internalized effort and/or arousal (for attentional dependence of CP, see Bidelman and Walker, 2017).

Here, we investigated how noise interference impacts cognitive load during perceptual identification of speech. Members of speech sound continua were presented in varying levels of noise to parametrically manipulate listening effort above and beyond that needed to classify unambiguous and ambiguous speech. Using pupillometry, we acquired continuous recordings of pupil dilation as a proxy of listening effort. If the grouping mechanisms of CP aid figure-ground perception of speech, we hypothesized unambiguous phonemes (categories) should elicit less noise-related changes in pupil responses than ambiguous tokens lacking a clear categorical identity. Our data show that the categorical nature of speech not only reduces cognitive load (listening effort) but also assists speech perception in noise degraded environments.

## Methods

### Participants

Fifteen young adults (3 males, 12 females; age: *M* = 24.3, *SD* = 1.7 years) from The University of Memphis participated in the experiment. All exhibited normal hearing sensitivity (i.e., <20 dB HL thresholds, 250–8000 Hz). Each participant was strongly right-handed (87.0 ± 18.2 laterality index; Oldfield, 1971) and had obtained a collegiate level of education (17.8 ± 1.9 years). Musical training enhances categorical processing and speech-in-noise listening abilities (Bidelman et al., 2014; Yoo and Bidelman, 2019). Consequently, all participants were required to have < 3 years of music training throughout their lifetime (mean years of training: 1.3 ± 1.8 years). All were paid for their time and gave written informed consent in compliance with a protocol approved by the Institutional Review Board at the University of Memphis.

### Speech Stimuli and Behavioral Task

We used a synthetic five-step vowel continuum previously used to investigate the neural correlates of CP (see Figure 1 of Bidelman et al., 2013; Bidelman and Walker, 2017). Each token was separated by equidistant linear steps acoustically based on first formant frequency (F1) yet was designed to be perceived categorically from /u/ to /a/. Although vowel sounds are perceived less categorically than other speech sounds (e.g., stop-consonants; Pisoni, 1973, 1975; Altmann et al., 2014), they do not carry intrinsic features upon which to make category judgments (formant transitions in consonants, for example, allow comparisons within the stimulus itself) (for discussion, see Xu et al., 2006). In contrast, steady-state features like the F1 contrast of our static vowels lack an intrinsic reference so categorical hearing of these stimuli necessarily requires acoustic features be matched to the best exemplar in long-term memory (Pisoni, 1975; Xu et al., 2006). Thus, we explicitly chose vowels because they more heavily tax perceptual-cognitive processing, and therefore listening effort, as might be revealed via pupillometry.

Tokens were 100 ms, including 10 ms of rise/fall time to reduce spectral splatter in the stimuli. Each contained identical voice fundamental (F0), second (F2), and third formant (F3) frequencies (F0: 150, F2: 1090, and F3: 2350 Hz). The F1 was parameterized over five equal steps between 430 and 730 Hz such that the resultant stimulus set spanned a perceptual phonetic continuum from /u/ to /a/ (Bidelman et al., 2013). Speech stimuli were delivered binaurally at 75 dB SPL through shielded insert earphones (ER-2; Etymotic Research) coupled to a TDT RP2 processor (Tucker Davis Technologies). This same speech continuum was presented in one of three noise blocks to vary SNR: unmasked, 0 dB SNR, −5 dB SNR. The masker was a speech-shaped noise based on the long-term power spectrum (LTPS) of the vowel set. While we typically use speech babble in our ERP studies, pilot testing showed this type of noise was too difficult for concurrent vowel identification, necessitating the use of simpler LTPS noise. The noise was presented continuously so that it was not time-locked to the stimulus presentation. Block order was randomized within and between participants.

During eyetracking, participants heard 150 trials of each speech token (per noise block). On each trial, participants labeled the sound with a binary response (“u” or “a”) as quickly and accurately as possible. Following a behavioral response, the interstimulus interval (ISI) jittered randomly between 800 and 1000 ms (20 ms steps, uniform distribution) before the next trial commenced. EEG was also recorded during the categorization task. These data are reported elsewhere (Bidelman et al., 2019b).

### Pupillometry Recording and Analysis

A Gazepoint GP3 eyetracker acquired listeners’ gaze fixations based on published procedures from our laboratory (Bidelman et al., 2019a). This device provides precise measurement of the location of ocular gaze and pupil diameter with an accuracy of ∼1° visual angle via an infrared, desktop mounted camera. In addition to cognitive effort, a number of factors affect pupillometry including the pupillary light reflex (Fan and Yao, 2011) produced by the sympathetic nervous system (Andreassi, 2000). Consequently, the sound booth’s lights remained off during the task. Participants could wear corrective lenses in the form of contacts. Continuous eye data were collected from the left and right eyes every 16.6 ms (i.e., 60 Hz sampling rate). MATLAB logged data from the GP3 via an API interface. Continued alignment with the screen was ensured by re-calibrating the eyetracker before each stimulus block. The GP3’s internal routine calibrated the eyes at nine-points across the horizontal/vertical dimensions of the screen.

Continuous eye data were recorded online while participants performed the auditory CP task. A central fixation cross-hair (+) remained on the computer screen during the auditory task to center and maintain participants’ gaze. Time stamps triggered in the data file demarcated the onset of each stimulus presentation. This allowed us to analyze time-locked changes in eye data for each stimulus akin to an evoked potential in the EEG literature (Beatty, 1982; Eckstein et al., 2017). Continuous recordings were filtered using a passband of 0.001–15 Hz, epoched [−100 to 1000 ms] (where *t* = 0 marks speech onset), baseline corrected, and ensemble averaged in the time domain to obtain the evoked pupil dilation response for each speech token per SNR and participant. This resulted in 15 waveforms per participant (= 5 tokens ^∗^ 3 SNRs). Blinks were automatically logged by the eye tracker and epochs contaminated with these artifacts were discarded prior to analysis. Additionally, to correct for subtle changes in the distance between the eyetracker camera and the participant that could affect pupil measurements (e.g., during head movement), the Gazepoint records a continuous scale factor for each pupil; a scale value = 1 represents pupil depth (distance to the camera) at the time of calibration, scaling < 1 reflects when the user is closer to the eyetracker, and a scaling > 1 when the user is further away. This scale factor was then used to weight the running time course prior to averaging and correct for movement artifacts.

### Data Analysis

#### Behavioral Data

Identification scores were fit with a sigmoid function *P* = 1/[1 + *e*^–^*^β1^*^(^*^*x*^*^–^*^β0^*^)^], where *P* is the proportion of trials identified as a given vowel, *x* is the step number along the stimulus continuum, and β*_0_* and β*_1_* the location and slope of the logistic fit estimated using non-linear least-squares regression. Larger β*_1_* values reflect steeper psychometric functions and stronger categorical perception. Behavioral speech labeling speeds (i.e., reaction times; RTs) were computed as listeners’ median response latency across trials for a given condition. RTs outside 250–2500 ms were deemed outliers (e.g., fast guesses, lapses of attention) and were excluded from analysis (Bidelman et al., 2013; Bidelman and Walker, 2017).

#### Pupillometry Data

To quantify the physiological data, we measured the peak (maximum) pupil diameter and latency within the search window between 300 and 700 ms. Visual inspection of the waveforms showed pupil responses were maximal in this timeframe (see Figure 2). Unless otherwise specified, dependent measures were analyzed using a two-way, mixed model ANOVA (subject = random factor) with fixed effects of SNR (three levels: unmasked, 0 and −5 dB SNR) and token [five levels: vw1-5] (PROC GLIMMIX, SAS^®^ 9.4; SAS Institute, Inc.). Tukey–Kramer and Bonferroni adjustments were used to correct subsequent *post hoc* and planned multiple comparisons, respectively.

## Results

### Behavioral Data

**FIGURE 1:**
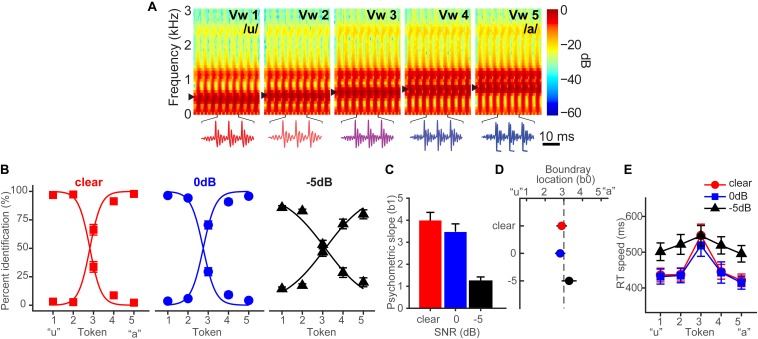
Spectrograms and behavioral speech categorization at three levels of signal-to-noise ratio (SNR). **(A)** Spectrograms of individual speech tokens. **(B)** Perceptual psychometric functions. Note the curves are mirror symmetric reflecting the percentage of “u” (left curve) and “a” identification (right curve), respectively. **(C)** Slopes and **(D)** locations of the perceptual boundary show that speech categorizing is robust even down to 0 dB SNR. **(E)** Speech classification speeds (RTs) show a categorical slowing in labeling (Pisoni and Tash, 1974; Bidelman and Walker, 2017) for ambiguous tokens (midpoint) relative to unambiguous ones (endpoints) in unmasked and 0 dB SNR conditions. Categorization accuracy and speed deteriorate with noise interference by remains possible until severely degraded SNRs. Data reproduced from Bidelman et al. (2019b). Spectrogram reproduced from Bidelman et al. (2014), with permission from John Wiley & Sons. errorbars = ± SEM.

Bidelman et al. (2019b) fully describes the behavioral results. Figure 1A shows spectrograms of the individual speech tokens and Figure 1B shows behavioral identification functions across the SNRs. An analysis of slopes (β*_1_*) revealed a main effect of SNR [*F*_2_,_28_ = 35.25, *p* < 0.0001] (Figure 1C). *Post hoc* contrasts confirmed that while 0 dB SNR did not alter psychometric slopes relative to unmasked speech (*p* = 0.33), the psychometric function became shallower with −5 dB SNR relative to 0 dB SNR (*p* < 0.0001). Additionally, SNR marginally but significantly shifted the perceptual boundary [*F*_2_,_28_ = 5.62, *p* = 0.0089] (Figure 1D). Relative to unmasked speech, −5 dB SNR speech shifted the perceptual boundary rightward (*p* = 0.011), suggesting a small but measurable bias to report “u” (i.e., more frequent vw1-2 responses) when noise exceeds the signal. Collectively, these results suggest that categorical representations are largely resistant to acoustic interference until signal strength of noise exceeds that of speech.

Behavioral response times (RTs) show the speed of categorization (Figure 1E). RTs varied with SNR [*F*_2_,_200_ = 11.90, *p* < 0.0001] and token [*F*_4_,_200_ = 5.36, *p* = 0.0004]. RTs were similar for unmasked and 0 dB SNR speech (*p* = 1.0) but slower for −5 dB SNR (*p* < 0.0001). *A priori* contrasts revealed this slowing was most prominent for more categorical tokens (vw1-2 and vw4-5). Ambiguous tokens (vw3) elicited similar RTs across noise conditions (*p*s > 0.69), suggesting that noise effects on RT were largely restricted to accessing categorical representations, not general slowing of decision speed across the board. We examined whether conditions elicited customary slowing in RTs near the midpoint of the continuum (Pisoni and Tash, 1974; Poeppel et al., 2004; Bidelman et al., 2013). Planned contrasts revealed this CP hallmark for unmasked [mean(vw1,2,4,5) vs. vw3; *p* = 0.0003] and 0 dB SNR (*p* = 0.0061) conditions, but not at −5 dB SNR (*p* = 0.59).

### Pupillometry Data

**FIGURE 2:**
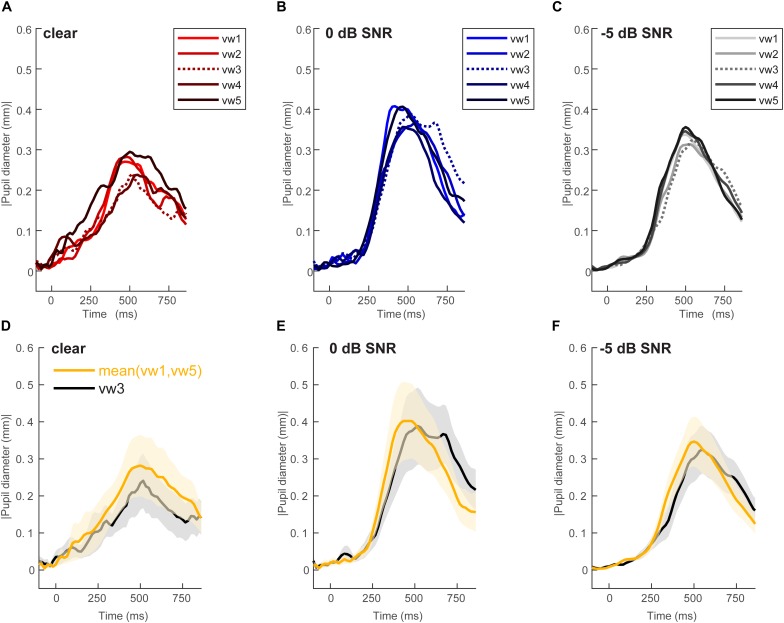
Grand average waveforms for pupil responses. Average responses to each token condition at each SNR level: **(A)** unmasked, **(B)** 0 dB SNR, **(C)** –5 dB SNR conditions. Peak pupil diameter and latency between the 300 and 700 ms search window are extracted for further analysis. Grand average waveforms for pupil responses contrasting categorical [mean (vw1,vw5)] vs. ambiguous (vw3) tokens at each SNR level. **(D)** Unmasked, **(E)** 0 dB SNR, **(F)** –5 dB SNR conditions. Pupil responses are modulated by SNR and token identity. shading = 1 SEM.

**FIGURE 3:**
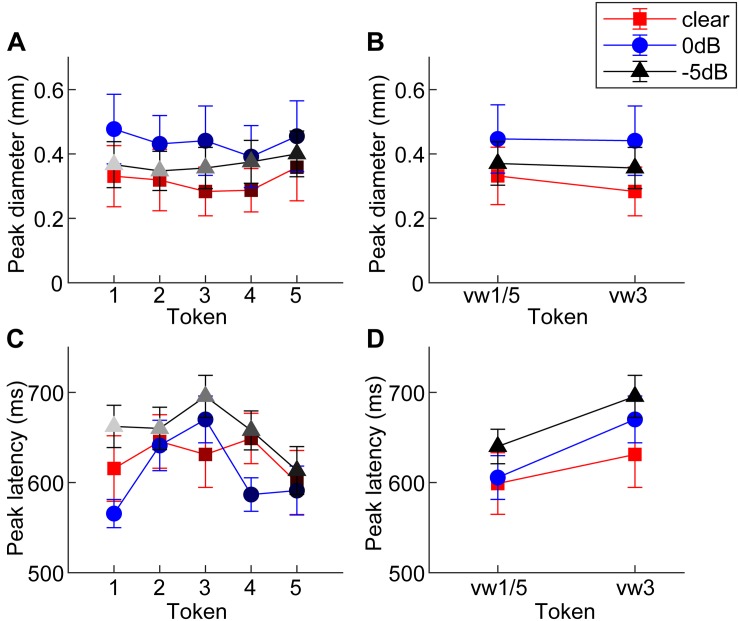
Mean peak pupil diameters and latencies by SNR. **(A)** Larger pupil size is observed at 0 dB SNR relative to unmasked and –5 dB SNR. **(B)** Peak pupil diameter is elevated at 0 dB SNR relative to the other two conditions. **(C,D)** In general, –5 dB speech shows the longest peak latencies of the three conditions. Pupil responses are delayed for 0 dB SNR speech and for categorically ambiguous speech (i.e., vw3 > vw1/5). errorbars = 1 SEM.

Figure 2 shows grand average pupil waveforms for each speech token and SNR as well as the responses specifically contrasting unambiguous [mean (vw1,vw5)] vs. ambiguous (vw3) tokens. Visually, the data indicated that both SNR and the categorical status of speech modulated pupil responses. To quantify these effects, we pooled the peak (maximum) pupil diameter and latency of unambiguous tokens (vw1 and vw5) (those with stronger category identities) and compared them with the ambiguous vw3 token (Liebenthal et al., 2010; Bidelman, 2015; Bidelman and Walker, 2017). Figure 3 shows the mean peak pupil diameters and latencies by SNR and behavioral RTs.

An ANOVA revealed a sole main effect of SNR on peak pupil size [*F*_2_,_196_ = 6.69, *p* = 0.0015] with no token [*F*_4_,_196_ = 0.53, *p* = 0.7157] nor token^∗^SNR interaction effect [*F*_8_,_196_ = 0.16, *p* = 0.9959] (Figure 3A). Planned contrasts of pupil size between pairwise SNRs showed that only unmasked speech differed from intermediate SNR speech. Specifically, pupil diameter increased when classifying speech in moderate interference (i.e., 0 dB > unmasked; *p* = 0.0007) but did not differ with further increases in noise level (i.e., 0 dB = −5 dB; *p* = 0.0794) (Figure 3B).

An ANOVA on pupil latency revealed that SNR strongly modulated pupil response timing [*F*_2_,_196_ = 4.60, *p* = 0.0112], as did whether the token was unambiguous [*F*_4_,_196_ = 3.25, *p* = 0.0130] (Figures 3C,D). There was not a token^∗^SNR interaction effect [*F*_8_,_196_ = 0.94, *p* = 0.4827]. Follow-up contrasts revealed similar latencies for unmasked and 0 dB speech (*p* = 0.5379), but longer latencies at −5 dB relative to 0 dB speech (*p* = 0.0061). Paralleling the RT data, *a priori* contrasts revealed an “inverted V-shaped” pattern analogous to the behavioral data—a slowing in response timing for ambiguous relative to unambiguous tokens in the 0 dB SNR [mean(vw1,2,4,5) vs. vw3; *p* = 0.0244]. Unmasked and −5 dB speech did not exhibit this pattern (*ps* > 0.27).

**FIGURE 4:**
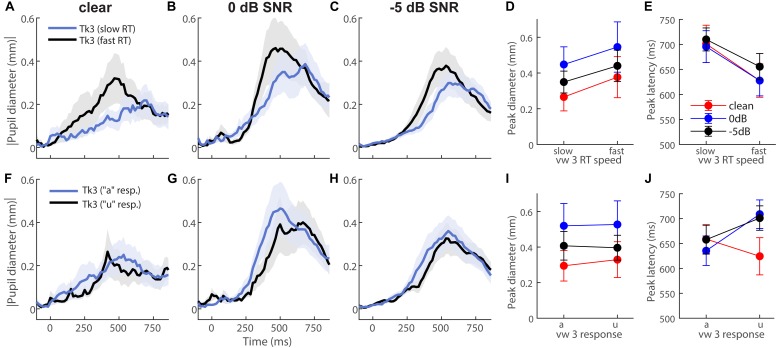
Pupil response latency but not size depends on speed of listeners’ decision. Grand average waveforms for pupil responses to vw3 based on **(A–E)** a median split of behavioral RTs and **(F–J)** the reported vowel category (e.g., “a” vs. “u”). **(E)** Pupil latencies strongly depend on speed of listeners’ decision. Slow RTs are associated with slower pupil responses to ambiguous token. **(D)** Pupil size is not dependent on RTs. **(I)** SNR has a sole effect on pupil response magnitudes when split by listeners’ identification (i.e., reporting vw3 as “u” vs. “a”). Pupil responses are again largest for 0 dB SNR speech compared to other noise conditions.

To further test whether behavior modulated eye behavior, we analyzed each listener’s single-trial vw3 pupil responses based on (i) a median split of their behavioral RTs into fast and slow responses (Figures 4A–E) and (ii) the vowel category they reported (e.g., “a” vs. “u”) (Figures 4F–J). This resulted in ∼75 trials for each subaverage. Despite having been elicited by an identical (though perceptually bistable) acoustic stimulus, vw3 pupil latencies were strongly dependent on the speed of listeners’ decision [*F*_1_,_70_ = 6.74, *p* = 0.0115]. Slow RTs were associated with slower pupil responses to the ambiguous token (Figure 4E). Pupil size was not dependent on RTs [SNR, speed, and SNR × speed effects: *ps* ≥ 0.0585] (Figure 4D). Split by listeners’ identification (i.e., vw3 reported as “u” vs. “a”), we found a sole main effect of SNR on pupil response magnitudes [*F*_2_,_70_ = 3.78, *p* = 0.0275]. Pupil responses were again largest for 0 dB SNR speech compared to the other noise conditions (Figure 4I). These data reveal that under similar states of speech ambiguity, pupil responses are modulated according to the speed of listeners’ behavioral categorization. Note, this contrasts EEG findings for the same stimuli, which show that electrical brain activity differentiates the ambiguous speech depending on listeners’ subjective report (i.e., vw3 heard as “u” vs. “a”) (Bidelman et al., 2013).

## Discussion

By recording continuous pupil responses during a rapid speech categorization task in noise, we assessed how acoustic interference impacts cognitive load and perceptual identification of phonemes. Our analyses revealed that speech perception was robust to moderate acoustic interference (i.e., ≥ 0 dB SNR). More category representative (less ambiguous) phonetic tokens reduced listening effort and were more resilient to moderate acoustic interference. While noise impacts perception of ambiguous phonemes, categorical coding appears to mitigate interference by enhancing representations of phonemes. We propose that categorical coding (i.e., speech with an unambiguous identity) helps partially counteract the negative effects of noise on perception, but only to the extent that speech signals are not too severely degraded. Our findings converge with notions that the process of categorization aids the extraction of speech from noise whereby abstract categories help fortify the speech code and make it more resistant to external noise interference (e.g., Helie, 2017; Bidelman et al., 2019b).

Physiologically, our data suggest that difficulty of speech processing modulates pupil behavior, but not straightforwardly. It is a common finding that pupil size increases when tasks are difficult to perform (Beatty, 1982). Consistent with our predictions, pupil size increased for moderately corrupted relative to unmasked speech but plateaued for severely corrupted speech. Previous work has assessed the pupil response to speech (sentences) across a broad range of intelligibility levels [i.e., −36 to −4 dB in nine 4 dB steps] (Zekveld and Kramer, 2014). This work suggests that pupil dilation increases at intermediate SNRs, but minimally at low and high SNRs, which has been interpreted to reflect intelligibility and/or task difficulty (Ohlenforst et al., 2017). The fact that pupil diameter of our participants increased with moderate SNR suggests the task demands in this condition did not exceed available cognitive resources. A recent pupillometry study found that pupil behavior correlates with subjective ratings of salience defined in terms of how noticeable or remarkable sounds are considered, indicating greater listening demand or arousal (Liao et al., 2016). In this vein, our result might reflect a performance/arousal tradeoff known as Yerkes-Dodson law, a phenomenon where performance resembles an inverted-U function of arousal (Yerkes and Dodson, 1908). Pupil dilation correlates with arousal responses measured in the locus coeruleus (LC) (Aston-Jones and Cohen, 2005). A variety of cognitive tasks elicit a strong relationship between performance and LC activity, whereby activation in the middle of the Yerkes-Dodson curve is associated with increased performance and task engagement (for reviews, see Berridge and Waterhouse, 2003; Aston-Jones and Cohen, 2005; Sara and Bouret, 2012). Under this framework, listeners are less attentive and disengaged (hypoarousal) and thus perform more poorly; when LC activity increases beyond intermediate range, listeners would be more distracted (hyperarousal), which would also reduce performance. Interestingly, a neuroimaging study reported a similar finding in neural responses over left temporal cortex and premotor cortex, with greater activity for slightly degraded speech relative to unmasked and severely degraded speech (Davis and Johnsrude, 2003), paralleling our pupillometry results.

The most interesting findings were for pupil latency. Previous work has shown that reduced speech intelligibility systematically delays pupil responses (Zekveld et al., 2010), implying increased listening effort. While we found responses were more delayed at severe than intermediate noise levels, latencies for unmasked and intermediate speech did not differ overall (i.e., unmasked = 0 dB). Listeners may have compensated by exerting more effort in the intermediate noise condition (McGarrigle et al., 2017). Importantly, pupil responses were more categorical at intermediate SNRs, as evidenced by a slowing in pupil responses for ambiguous tokens. This pattern was not observed at −5 dB SNR. These findings suggest categorical coding helps reconstruct degraded speech sounds with unambiguous identities, but only within a limited range of intelligibility.

Behaviorally, psychometric slopes were steeper for unmasked relative to noise-degraded speech, and only became flatter for severely degraded speech. Indeed, only highly degraded speech weakened CP, further suggesting that the natural binning process of categorical coding helps maintain robust perception of SIN. Presumably, CP enhances processing within the acoustic space to help phonetic representations stand out (e.g., Nothdurft, 1991; Perez-Gay et al., 2018). We argue that noise-related decrements in CP reflect weakening of internalized categories rather than less vigilant listening across the board because ambiguous tokens elicited similar RTs across noise levels. Moreover, both our behavioral and physiological data indicated more categorical responses to unambiguous relative to ambiguous tokens at intermediate noise levels. Thus, noise-related decrements in our data likely reflect fuzzier matches between speech signals and templates of speech sounds (Bidelman et al., 2019b).

Discrepancies between the behavioral and physiological data in SNR which showed categorical coding (i.e., inverted-V pattern) suggest perhaps that pupil responses are less sensitive than behavior and require the additional “load” of intermediate noise to show a categorical effect in response timing. Additionally, while the −5 dB condition produced significantly worse behavioral performance relative to quiet, it was the 0 dB condition instead that produced larger peak pupil dilation. This could reflect the fact that the 0 dB condition was more effortful than quiet, despite behavioral accuracy remaining high. Such findings align with notions of the FUEL model (Pichora-Fuller et al., 2016) suggesting performance is governed by a combination of signal quality (e.g., input SNR) and internal factors (e.g., arousal, attention, and motivation).

One interpretation of CP is that ambiguous or intermediate tokens are “drawn” toward prototypes or category centers, i.e., the veridical percept is warped by the existence of a category representation such that peripheral tokens are perceived as more central (e.g., “perceptual magnet” theory; Kuhl, 1991; Iverson et al., 2003). Our physiological data loosely align with this notion, showing and influence of category prototypicality/centrality on degraded speech perception. Peripheral tokens (e.g., vw2 and vw4) elicited similar pupil responses to their central prototype (i.e., continuum endpoints), as evidenced by the inverted-V pattern in RT (Figure 1E) and pupil latency data (Figure 3C). Still, for speech sounds which split the perceptual boundary (i.e., vw3)—and are thus perceptually ambiguous—we find this perceptual draw is considerably weaker if made at all. This is supported by the fact pupil responses to vw3 were similar when split by listeners’ subjective report (“u” vs. “a”; Figures 4F–H). Collectively, these later findings align with more relaxed models of perception which consider gradiency, whereby the system must balance the efficiency of discarding potential rich and continuous acoustic details with discrete category representations (McMurray et al., 2008). Thus, one might equally discuss our findings as reflecting the gradience of phonetic categories (especially vowels), and more generally perceptual uncertainty, rather than CP *per se.* Under this interpretation, acoustic cues that allow the rapid assessment of category membership of unambiguous tokens (e.g., vw1, vw5) are acoustically/perceptually available until noise masking is too egregious. In cases in which speech cues are ambiguous (vw3), noise fails to alter the decision process much, because listeners are already dealing with ambiguous acoustic-phonetic information.

Collectively, our findings converge with notions that categorical representations of phonemes are more salient and resilient to noise degradation than acoustic-sensory ones (Helie, 2017; Bidelman et al., 2019b, c). On the premise that phonetic representations (a high-level code) are more resilient to noise than surface level features (a low-level code) (Helie, 2017; Bidelman et al., 2019b, c), the construction of perceptual objects and natural binning process of CP might mitigate noise by helping category members stand out among a noisy feature space. Despite being acoustically dissimilar, categorically equivalent sounds would elicit similar changes in local firing rate, whereas cross-category (perceptually distinct) sounds would not (e.g., Recanzone et al., 1993; Guenther and Gjaja, 1996; Guenther et al., 2004). Noise would create a noisier map for physical acoustic details, but phonetic categories would persist (e.g., Nothdurft, 1991; Perez-Gay et al., 2018).

We found that ambiguous speech increased listening effort (delayed pupil responses). Results from fMRI similarly suggest that activation of auditory cortical cells may be shorter for category prototypes than for other sounds (Guenther et al., 2004). Indeed, participants labeled unambiguous tokens more quickly than ambiguous tokens, suggesting more efficient processing of members from well-formed categories. This advantage was also observed in pupil latencies in the intermediate noise condition, but not in the unmasked condition. Delayed pupil responses might instead reflect processes of ambiguity resolution. In speech, there is no one-to-one correspondence between any single acoustic cue and phonetic representations (Lotto and Holt, 2016). Partial loss of acoustic cues would render phonemes highly confusable with one another. Connectionist models of speech perception such as TRACE (McClelland and Elman, 1986) posit bi-directional, interactive activation of phonemic traces that help recover meaning when signal features are missed. Under TRACE, speech processing transpires through a neuronal network representing speech features at increasingly higher levels. Incoming acoustic input activates nodes for features (and inhibits others), which in turn activate phonemes at the next level. During this process, traces of inhibited representations remain activated for a period, helping the listener recover information if errors are perceived (e.g., missing an acoustic segment). If noise leads to partial loss of cues, delayed pupil responses observed in our data might reflect ongoing activation (through a TRACE-like network) of multiple phonetic representations in attempt to disambiguate what is being heard.

In sum, the present findings demonstrate that pupillometry can be used as an effective technique for assessing underlying processes of speech perception and categorical processing. Here, the benefits of tracking CP with pupillometry were twofold: (a) providing complementary physiological data for comparison with existing data, and (b) lending temporally sensitive insight into mental processes not available from behavioral measures alone.

## Data Availability Statement

The datasets generated for this study are available on request to the corresponding author.

## Ethics Statement

The studies involving human participants were reviewed and approved by The University of Memphis IRB. The participants provided their written informed consent to participate in this study.

## Author Contributions

Both authors listed have made a substantial, direct and intellectual contribution to the work, and approved it for publication.

## Conflict of Interest

The authors declare that the research was conducted in the absence of any commercial or financial relationships that could be construed as a potential conflict of interest.
