# Corrigendum: Autonomic Nervous System Correlates of Speech Categorization Revealed Through Pupillometry

**DOI:** 10.3389/fnins.2020.00132

**Published:** 2020-02-19

**Authors:** Gwyneth A. Lewis, Gavin M. Bidelman

**Affiliations:** ^1^Institute for Intelligent Systems, The University of Memphis, Memphis, TN, United States; ^2^School of Communication Sciences and Disorders, The University of Memphis, Memphis, TN, United States; ^3^Department of Anatomy and Neurobiology, University of Tennessee Health Sciences Center, Memphis, TN, United States

**Keywords:** pupillometry, categorical perception, speech-in-noise (SIN) perception, listening effort, eye behavior

In the original article, there was an error in the text. The methods described the noise masker as a four-talker babble (three women and one man) extracted from the clinical QuickSIN test (e.g., Killion et al., 2004), which was incorrect. The masker was actually a speech-shaped noise based on the long-term power spectrum.

A correction has been made to the *METHODS* section, subsection *Speech Stimuli and Behavioral Task* paragraph 2:

Tokens were 100 ms, including 10 ms of rise/fall time to reduce spectral splatter in the stimuli. Each contained identical voice fundamental (F0), second (F2), and third formant (F3) frequencies (F0: 150, F2: 1090, and F3: 2350 Hz). The F1 was parameterized over five equal steps between 430 and 730 Hz such that the resultant stimulus set spanned a perceptual phonetic continuum from /u/ to /a/ (Bidelman et al., 2013). Speech stimuli were delivered binaurally at 75 dB SPL through shielded insert earphones (ER-2; Etymotic Research) coupled to a TDT RP2 processor (Tucker Davis Technologies). This same speech continuum was presented in one of three noise blocks to vary SNR: unmasked, 0 dB SNR, −5 dB SNR. The masker was a speech-shaped noise based on the long-term power spectrum (LTPS) of the vowel set. While we typically use speech babble in our ERP studies, pilot testing showed this type of noise was too difficult for concurrent vowel identification, necessitating the use of simpler LTPS noise. The noise was presented continuously so that it was not time-locked to the stimulus presentation. Block order was randomized within and between participants.

The authors apologize for this error and state that this does not change the scientific conclusions of the article in any way. The original article has been updated.
